# Establishment of triple-negative breast cancer cells based on BMI: A novel model in the correlation between obesity and breast cancer

**DOI:** 10.3389/fonc.2022.988968

**Published:** 2022-12-14

**Authors:** Daniela Shveid Gerson, Raquel Gerson‐Cwilich, Cesar Octavio Lara Torres, Alberto Chousleb de Kalach, José Luis Ventura Gallegos, Luis Ernesto Badillo‐Garcia, Juan Enrique Bargalló Rocha, Antonio Maffuz‐Aziz, Ernesto Roberto Sánchez Forgach, Gerardo Castorena Roji, Carlos D. Robles Vidal, Ariana Vargas‐Castillo, Nimbe Torres, Armando R. Tovar, Mariela Contreras Jarquín, Jesús Tenahuatzin Gómez Osnaya, Alejandro Zentella‐Dehesa

**Affiliations:** ^1^ Cancer Center, American British Cowdray (ABC) Medical Center, Mexico City, Mexico; ^2^ Pathology Department, Instituto Nacional de Ciencias Médicas y Nutrición Salvador Zubirán (INCMNSZ), Mexico City, Mexico; ^3^ Experimental Surgery Department, American British Cowdray (ABC) Medical Center, Mexico City, Mexico; ^4^ Department of Genomic Medicine and Environmental Toxicology, Institute of Biomedical Research, National Autonomous University of Mexico, Mexico City, Mexico; ^5^ Biochemistry Unit, Instituto Nacional de Ciencias Médicas y Nutrición Salvador Zubirán (INCMNSZ), Mexico City, Mexico; ^6^ Department of Nutrition Physiology, Instituto Nacional de Ciencias Médicas y Nutrición Salvador Zubirán (INCMNSZ), Mexico City, Mexico

**Keywords:** breast cancer, obese adipose tissues, BMI – body mass index, triple negative breast cancer, cell lines, cell culture, leptin and endothelial activation

## Abstract

**Introduction:**

Obesity has been associated with an increased risk of biologically aggressive variants in breast cancer. Women with obesity often have tumors diagnosed at later stages of the disease, associated with a poorer prognosis and a different response to treatment. Human cell lines have been derived from specific subtypes of breast cancer and have served to define the cell physiology of corresponding breast cancer subtypes. However, there are no current cell lines for breast cancer specifically derived from patients with different BMIs. The availability of those breast cancer cell lines should allow to describe and unravel functional alterations linked to these comorbidities.

**Methods:**

Cell cultures were established from tumor explants. Once generated, the triple negative subtype in a patient with obesity and a patient with a normal BMI were chosen for comparison. For cellular characterization, the following assays were conducted: proliferation assays, chemo – sensitivity assays for doxorubicin and paclitaxel, wound healing motility assays, matrix invasion assays, breast cancer cell growth to estradiol by chronic exposure to leptin, induction of endothelial permeability and tumorigenic potential in athymic mice with normo - versus hypercaloric diets with an evaluation of the epithelium – mesenchymal transformation proteins.

**Results:**

Two different cell lines, were established from patients with breast cancer: DSG-BC1, with a BMI of 21.9 kg/m2 and DSG-BC2, with a BMI of 31.5 kg/m2. In vitro, these two cell lines show differential growth rates, motility, chemosensitivity, vascular permeability, response to leptin with an activation of the JAK2/STAT3/AKT signaling pathway. In vivo, they displayed distinct tumorigenic potential. In particular, DSG-BC2, presented higher tumorigenicity when implanted in mice fed with a hypercaloric diet.

**Discussion:**

To our knowledge, these primary cultures are the first in vitro representation of both breast cancer and obesity. DSG – BC2 presented a more aggressive in vivo and in vitro phenotype. These results support the hypothesis that breast cancer generated in an obese metabolic state may represent a contrasting variant within the same disease. This new model will allow both further comprehension, functional studies and the analysis of altered molecular mechanisms under the comorbidity of obesity and breast cancer.

## Introduction

In Mexico, cancer represents the third cause of mortality in women over 40 years of age (1, 2). Among them, breast cancer is the first cause of female mortality associated with neoplastic diseases and hence considered an emergent public health problem (3, 4). This mortality is associated with metastatic invasion of vital organs such as the lung, bone, liver, and brain. Breast cancer incidence and mortality are associated subsequently with two important aspects with regards to the Mexican population: age and overweight. The inversion of the population pyramid predicts an increase in the number of women over 50 years of age in the upcoming decades (5) and an increase in the frequency of obesity and overweight in more than 70% of women in our country (6, 7). In fact, Mexico is considered the country with the highest obesity rates worldwide.

The incidence of breast cancer in obese women is up to three times greater compared to that in women with ideal body weight (8) and is associated with particular biological characteristics. Studies have demonstrated that patients with obesity and cancer have a lower global survival and greater possibility of cancer recurrence. This in turn is caused by hormones involved in obesity, such as adipokines, glucocorticoids, and insulin, secreted in an abnormal fashion and acquire aberrant signaling promoting fat storage (9). This further propagates obesity and an increased production of such hormones, contributing to the development of numerous diseases, among which cancer is highly prevalent. Taking this into consideration, it is important to study whether breast cancer in obese women differs regarding biology and therapeutic susceptibility.

In Mexico, there is but one study that links overweight and obesity as adverse prognostic factors in breast cancer (10, 11). Obesity has been related to multiple cancer subtypes due to the chronic state of inflammation contained in the adipose tissue, the increment in circulating levels of insulin, and the increase in insulin receptors, which favor autonomous growth and alterations in adipokines, hormones, and metabolites associated to epigenetic changes. The systemic changes induced by obesity generate a particular tumorigenic effect, cellular proliferation, cancer progression, and subsequent drug resistance and cancer recurrence (12).

In relation to breast cancer, obesity has been associated with an increased risk of more biologically aggressive variants, notably triple-negative breast cancer. It also promotes invasion and metastasis (13). Women with obesity often have tumors diagnosed at later stages of the disease, which, aside from immuno-phenotype and stage, is thus associated with a poorer prognosis and a different response to treatment.

There are no current cell lines for breast cancer previously derived from patients with different BMI or specifically with an obese phenotype. We aimed to create cell cultures derived from patients with BMI <25and >30 kg/m^2^ to further investigate differences between the cellular biology and molecular profiles in breast cancer and obesity compared to breast cancer in women with ideal weight. We established two different cell lines, DSG-BC1 and DSG-BC2, derived from a woman with ideal weight and obesity, respectively, both triple-negative immune subtypes, and performed a preliminary biological characterization. To our knowledge, these primary cultures are the first *in vitro* representation where the influence of the comorbidity of breast cancer and obesity can be compared to breast cancer without overweight or obesity.

## Materials and methods

### Clinical characteristics

This research protocol was submitted to the research and ethics committee of The ABC Medical Center in Mexico City, approved with registration number ABD 14.04. Inclusion criterion for the study was the following: women with a previous biopsy with a pathological result of breast cancer at any stage, who were subjected to a surgical intervention for the removal of cancer from the breast, in which at least 5 mm^3^ of tissue was obtained. Once patients signed the informed consent, they were divided into three groups: BMI below 25 kg/m^2^, BMI between 25 and 30 kg/m^2^, and those with a BMI over 30 kg/m^2^. Exclusion criteria included breast cancer recurrence, inflammatory breast cancer or processes, breast trauma, and previous systemic therapy of any kind in relation to the current breast neoplasm. A total of 32 women participated in the study. They were between 37 and 73 years of age with an average age of 55. Of these women, 27 had ductal invasive breast cancer and 5 with lobular infiltrating carcinomas. Regarding their immune phenotype, there were seven triple-negative breast cancers; the rest were hormone positive, including four triple positives with an HER2 amplification. Six women were obese, 13 were overweight, and 13 had a BMI under 25 kg/m^2^. There were 13 patients who presented comorbidities including diabetes mellitus type II (4), hypertension (2), hypothyroidism (3), hypercholesterolemia (1), hiatal hernia (1), gastroesophageal reflux disease (1), and fibromyalgia (1).

### Establishment of cell cultures

**Figure 1:**
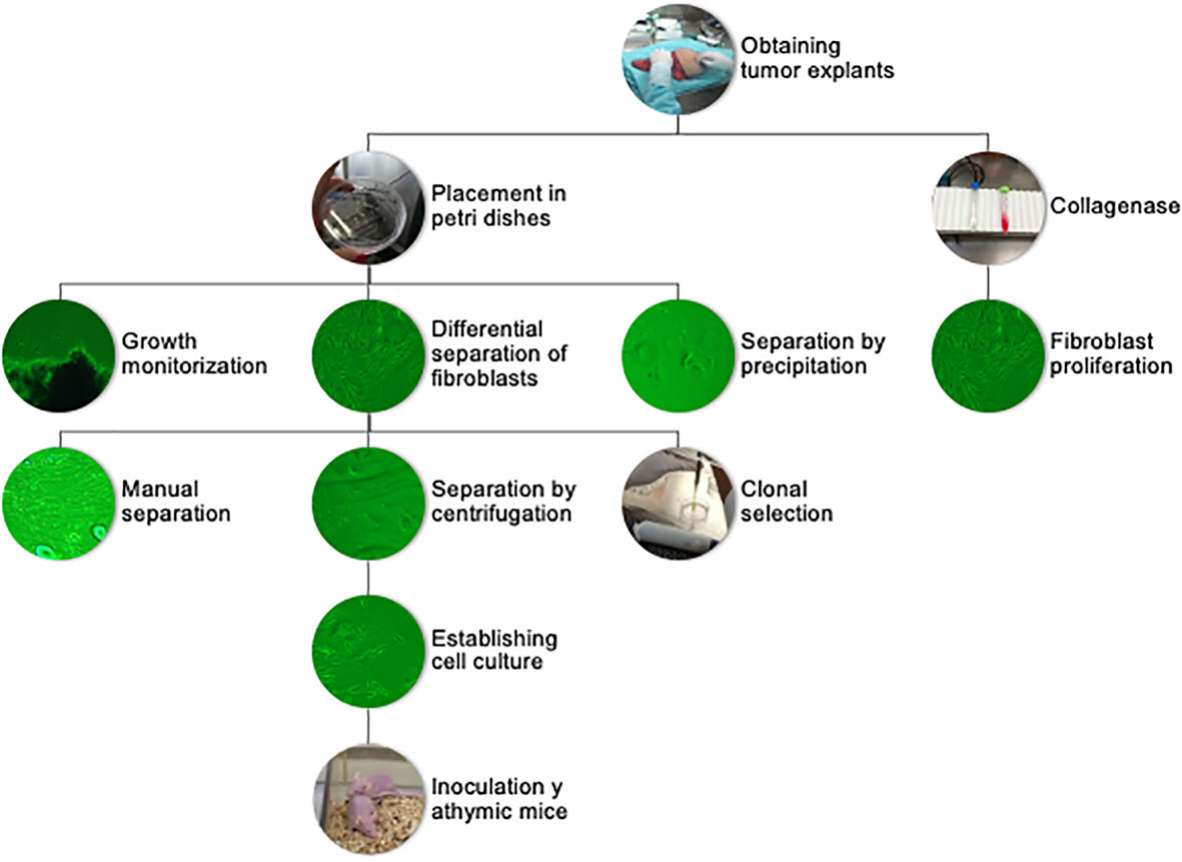
Flowchart of sample processing, from tumor explant to primary cultures and tumorigenic assay.

Patients were selected from the ABC Medical Center Cancer Center, and signature of the informed consent was obtained. Through a collaboration sub-protocol with surgical oncology, nursing, and pathology, we were able to receive tumor fragments of at least 5 mm^3^ from excised breast tissue derived from partial or total mastectomies. The tissue fragments were then placed in growth medium for transportation. Once in the sterile Hood, the medium was aspirated, and the explant was placed on sterile gauze to remove excess liquid. The explant was then transferred to a Petri dish where they were cut into approximately 1 mm × 1 mm pieces and spread throughout the dish. Finally, they were attached to the bottom of a 3-cm diameter Petri dish and left to dry for 5 min to assure firm adhesion. After this, 3 ml of growth medium was added, and micrographs of the attached explants were taken with a Primovert inverted microscope (Zeiss). This first image served to keep record of cellular sprouting and growth. Finally, the plates were placed inside an incubator at 37°C under an atmosphere with 5% CO_2_ and 100% humidity. Cell sprouting and growth were monitored daily. The first cells to spread and proliferate from the explants were fibroblasts with rapid proliferation rates. After approximately 3–6 weeks, tumor cells began to spread from the periphery of the explants. For isolation of these tumor cells, different procedures were evaluated, including the use of collagenase, manual scraping of fibroblast under the dissection microscope based on cell morphology, differential sedimentation rates, and selection of cellular populations using cloning rings. Manual separation and the selection of clonal populations were the most effective in isolating tumor cells and were the sole method used after the initial 10 explants (**Figure 1**). A total of 32 tumor explants were obtained. They were paired by histology and immunohistochemistry subtype and matched to have a pair with and without obesity. We were able to establish two cell cultures from patients with ductal invasive triple-negative breast cancer, one without obesity (DSG-BC1) and one with obesity (DSG-BC2).

### Proliferation assays

For direct cell count, 12,000 cell/well were plated in 48-well plates (Corning Costar), and every 24 h, the cells of three wells were detached with a Trypsin (0.25%)/EDTA (0.02%) solution in phosphate-buffered saline (PBS) (T4049 Sigma Aldrich, St. Louis, MO) and counted in a hemocytomer chamber by loading the two chambers with a 1:1 mixture v/v of the cell suspension with trypan blue solution. For indirect evaluation by measuring the increase in impedance, proliferation was evaluated indirectly by measuring electric impedance using the xCELLigence system. We plated 7,500 cells/well in a final volume of 200 μl/well in xCelligence 16-well E-plates (Cat No. 5469830001, Aligent, Santa Clara, CA). Every cell line was plated in triplicates. Impedance was measured every 30 min throughout 96 h. The cell division rate was calculated using the software provided with the xCelligence equipment and expressed as change in Cell Index/h.

### Chemo-sensitivity assays

Both breast cancer cell lines (DSG-BC-1 and DSG-BC-2) were plated in 48-well plates (Corning Costar) at a cell density of 12,000 cells/well. Three wells with cells remained without treatment, three other wells remained without cells, and all wells contained 500 μl growth medium. After 24 h, cells were exposed to increasing doses of paclitaxel or doxorubicin; 48 h later, the medium was removed by aspiration, and the remaining viable cells were fixed with 2% glutaraldehyde in growth medium for 30 min. The glutaraldehyde solution was removed, and all wells were stained with 250 μl of a solution with 0.5% crystal violet (Sigma Aldrich C0775, St. Louis, MO) in 20% methanol and 80% H_2_O v/v for 15 min, and excess satin was rinsed with tap water and allowed the plates to dry under air current. Crystal violet with 10% acetic acid solution was solubilized and read OD at 570 nm. The OD of the wells with cells was subtracted from the rest of the OD values. OD of the wells with cells but without chemotherapy served to assign 100% viability; the rest of the OD values were normalized and expressed as % viability.

### Wound healing motility assays

DSG-BC1 and DSG-BC2 cells were plated in 100 × 15 mm Petri dishes (Corning Costar) previously marked with lines in the exterior of the lower part of the Petri dish to guide the scratching. Cells were plated with growth medium at a cell density of 40,000 cells/cm^2^. The monolayers reached confluence within 24 h. The monolayers were scratched using a sterile pipette to generate a linear wound in eight different lines/plate. Cell motility was evaluated every 3 h; micrographs were taken for the eight scratched lines for each cell type. Using the image processing software Image J (public domain, Wayne Rasband NIH), the cell-free areas were outlined, and the surface was estimated in pixel units. Cell motility was evaluated as the reduction in cell-free area every 3 h and reported as average and standard deviation.

### Sensitization to E2 through chronic exposure to leptin

Sensitization to E2 was performed as previously described (14). Cells (2 × 10^3^) of the different breast cancer cell lines were plated and grown in Roswell Park Memorial Institute (RPMI)-1640 supplemented with 10% fetal bovine serum (FBS) and leptin (100 ng/ml). All the cell lines were grown at 37°C under a 5% CO_2_ atmosphere saturated with 100% H_2_O. After 4 days, the medium was replaced with phenol red-free RPMI-1640 supplemented with 10% delipidated FBS and leptin (100 ng/ml) for 24 h. Finally, the medium was replaced with phenol red-free RPMI-1640 supplemented with 5% delipidated FBS and leptin (100 ng/ml); cells were immediately challenged with 0, 10, and 100 nM E2. After 48 h of E2 treatment, cells were fixed and stained with crystal violet as described for the chemo-sensitivity assay.

### Preparation of tumor-cell-conditioned medium

Tumor-cell-conditioned medium was prepared as previously described (15). Briefly, each breast cancer cell line was cultured in 20 × 100 mm Petri dishes until it reached 80% confluence. After washing each plate 10 times with 10 ml of PBS/RPMI‐1640 (1:1 v/v) without phenol red (Laboratorios Microlab S.A. de C.V., D.F. Mexico, Mexico), the cells were maintained in 8 ml of serum-free RPMI without phenol red per plate. After 48 h incubation at 37°C under an atmosphere with 5% CO_2_ and 100% humidity, the culture medium was collected and lyophilized. The resulting powder was dissolved in distilled sterile water (1/10 of the original volume), dialyzed with a PM‐3 Ultrafiltration Membrane (EMD Millipore, Billerica, MA, USA), and sterilized using a 0.22-μm Millex‐GS syringe filter unit and supplemented with protease inhibitor cocktail (cOmpleteTM Protease Inhibitor Cocktail; Roche Applied Science, Indianapolis, IN, USA). The protein concentration was determined using the Bradford reagent assay (Bio-Rad, Hercules, CA, USA). The concentrated conditioned medium (CM) was kept at 4°C until use in a final dilution 1:10 v/v in growth medium.

### Evaluation of endothelial activation

Primary human endothelial cells derived from umbilical veins (HUVECs) were isolated from the veins of umbilical cords within 48 h of normal births as previously described (16). Briefly, the vein was canalized and perfused with PBS to remove blood clots from within. Once cleaned, it was sealed, and a 0.2% collagenase solution (Roche, cat. no. 103586, Basel SZ) prepared in PBS was infused and sealed. The cord was submerged in PBS at 37°C for 10 min and then gently massaged. Afterwards, one end was opened to recuperate dislodged endothelial cells in 50-ml test tubes, and the veins were perfused five times with 10 ml PBS. The mixture of endothelial cells, red blood cells, lymphocytes, and collagenase was centrifuged, and the pellet was reconstituted in endothelial growth medium: M199 medium (Thermo Fisher/GIBCO 11150067) supplemented with 10% FBS, glutamine (2mM), heparin (1 mg/ml) (Sigma Aldrich, St. Louis, MO), and endothelial mitogen (0.01 μg/ml) (Biomedical Technologies Inc., Stoughton, MA). Cells were plated in Petri dishes (Corning Costar); after 24 h, non-adherent cells were washed away with PBS, and HUVECs were cultured in endothelial growth medium and expanded for the next 2 weeks. Cells were detached from the plate with a Trypsin (0.25%)/EDTA (0.02%) solution in PBS (T4049 Sigma Aldrich, Saint Louis, MO); Endothelial growth medium was then added and centrifuged. The cell pellet was resuspended in endothelial growth medium and counted to plated 50,000 cell/cm^2^ for cell permeability assays or for adhesion of U937 cells.

### Adhesion of U937 cell to activated endothelial cells

A total of 50,000 HUVECs were plated/well in 24-well plates (Corning Costar) in endothelial growth medium. The cultures reached confluence after 24 h. After treating HUVECs for 3 h with a 1:10 dilution of the concentrated conditioned media from DSG-BC1 or DSG-BC2, the medium was removed, and fresh endothelial growth medium was added. U937 monocytes (1 × 10^6^) were pre-labeled overnight with 1 μCi/ml of 3H-thymidine (NEN, Boston, MA). A total of 250,000 pre-labeled U937 cells were added/well and co-incubated for 3 more hours. At the end of this period, non-adherent cells were removed, and the wells were gently washed with PBS. The radioactivity of the attached cells was counted after cell lysis with 500 μl 0.2 N NaOH and mixed with 3 ml of scintillation fluid (Ultima Gold LLT, Perkin Elmer, Waltham, MA). A triplicate of 250,000 pre-labeled U937 cells were set apart and counted in the scintillation counter. Radioactivity was estimated using a β-Counter (Beckman). The amount of label in adherent cells was normalized using the label of 250,000 pre-labeled cells as 100%.

### Changes in endothelial monolayer permeability

For the evaluation of HUVEC monolayer permeability, 12,000 HUVECs cells were plated in a 16-well xCelligence plate and placed in an incubator. When cells reached confluence, an initial reading was conducted to establish a baseline impedance using and RTCADP xCelligence equipment (Agilent, Santa Clara, CA). Afterwards, the conditioned medium was added, and impedance was recorded every 30 min throughout 116 h.

### Tumor-cell-conditioned medium

Tumor cells release a variety of bioactive protein factors and metabolites that can be collected in the medium conditioned by the tumor cells. We prepared tumor-cell-conditioned media from DSG-BC1 or DSG-BC2 as previously described (17). Both cell lines were plated at a cell density of 5 × 10^4^ cell/cm^2^ in 10-mm Petri dishes with 10 ml DMEM/F12K supplemented with 10% FBS and allowed to reach 80%–90% confluency. At that point, the medium was removed, and the cell monolayers were washed five times with 5 ml PBS to remove excess FBS. The cultures were fed with serum-free DMEM/F12K and incubated for 48 h at 37°C with a 5% CO_2_ atmosphere saturated with H_2_O. Cell viability was evaluated every 12 h looking for mitotic figures and increase in cell number. After 48 h, the medium was collected and centrifuged at 1,500 rpm for 6 min to remove floating cells. The supernatant was collected and kept 4°C until used in the invasion assay.

### Invasion assay

The colorimetric QCM 24-well collagen-based cell invasion assay (Merck ECM508) was used following the vendor’s instructions. Briefly, triplicates with 0.25 × 10^6^ cells/0.25 ml of growth medium (DMEM-F12K supplemented with 10% FBS) were seeded in the upper chambers; in the lower chambers, we added 500 μl of DMEM-F12K supplemented with 10% FBS and incubated at 37°C with a 5% CO_2_ atmosphere saturated with H_2_O. After adhesion for 8 h, the medium in the upper chamber was removed and replaced with growth medium without serum for further 12 h. The assay was started by placing different attractants in the lower chamber: growth medium with 10% FBS, growth medium with 20% FBS, growth medium with phorbol-ester-myristate (PMA) 80 nM final concentration or a 1:1 dilution v/v of serum-free medium conditioned by DSG-BC1 or DSG-BC2 in serum-free growth medium. After 24 h, the cells on both sides of the membrane were fixed and stained following the vendors protocol. Cells from the upper surface of the membrane were removed, and the stain from the cells in the lower surface of the filter was extracted and optical density quantified at 599 nm. OD from the wells with growth medium with 10% FBS was used as controls with 100% invasion.

### Soft agar colony formation assay

Colony formation was evaluated using a Nobel-soft system as previously described (18). Briefly, in 12-well plates, a two-layer system with 0.6% and 0.3% agarose content was prepared with 2× serum-free growth medium (DMEM-F12K). The second layer contained 2,500 cells/well. The spaces between the wells were filled with sterile water to reduce desiccation and the plates were incubated at 37°C with a 5% CO_2_ atmosphere saturated with H_2_O for 3 weeks. Micrographs were taken every 2 days to follow the emergence and growth of 3D colonies. Within the first week, colonies were detected in HeLa cells, which were used in this assay as positive controls.

### Cytoplasmic and nuclear extracts

Cytoplasmic and nuclear cell extracts were prepared as previously described (16) from two 10-mm Petri dishes with cell cultures at a confluence of 70%–80%. After scraping the cells in 1 ml PBS, they were subjected to centrifugation at 4,000 rpm for 5 min in a microfuge (Thermo Scientific). The supernatant was removed and the cell pellet frozen by placing the tubes in dry ice with ethanol. Cells were broken by adding 500 μl of a hypotonic buffer [10mM HEPES, pH 7.9, 10mM KCl, 1.5mM MgCl_2_, 1mM dithiothreitol (DTT), and 0.5mM phenylmethylsulfonyl fluoride (PMSF)] and subjected to centrifugation at 4,000 rpm for 5 min. The supernatant was considered to be enriched with cytoplasmic fraction.

Nuclei were gently resuspended in a hypertonic buffer (20mM HEPES, pH 7.9, 400mM NaCl, 1.5mM MgCl_2_, 0.2mM EDTA, 25% glycerol, 1mM DTT, and 0.5mM PMSF) and incubated in a rotating mixer (SOL BAT Aparatos Científicos) for 30 min. At the end of this incubation, nuclei were centrifuged at 14,000 rpm for 10 min; the supernatant was considered as the nuclear extract. Both extracts were diluted 1:1 con radioimmunoprecipitation assay (RIPA) buffer [150 mM NaCl, 10mM Tris–HCl, 1 mM EDTA, 1% Triton X-100, 0.1% sodium dodecyl sulfate (SDS), and 0.1% Na deoxycholate] supplemented with protease inhibitors (Complete, Roche). Protein concentration was estimated with Bradford reagent in both extracts (BioRad), and equal amounts of protein were diluted in sample buffer to a 1× final concentration (125mM Tris–HCl, pH 6.8, 1% SDS, 10% glycerol, 0.1% bromophenol blue, and 2% 2β-mercaptoethanol).

### Western blot

Western blot analysis was performed as previously described (19). Protein content from the cytoplasmic and nuclear extracts were separated using SDS polyacrylamide gel electrophoresis (SDS-PAGE) gels, 7.5% acrylamide for cytoplasmic extracts and 10% for nuclear extracts. Once resolved by electrophoresis, the proteins were transferred to polyvinylidene fluoride (PVDF) immobilon-P membranes (Millipore) and blocked with 5% fat-free milk powder in Tris-buffered saline (TBS) overnight at 4°C. After removing the blocking solution and washing the membranes with TBS, primary antibodies (Santa Cruz Biotechnology) were added diluted in TBS overnight at 4°C: anti-E-cadherin 1:250 (sc-8426), anti-vimentin 1:16,000 (sc-6260), anti-Ep-CAM 1:250 (sc-25308), and anti-β-actin 1:10,000 (sc-47778) for the cytoplasmic extracts, and anti-ZEB-1 1:250 (sc-515797), anti-Nanog 1:250 (sc-293121), and anti-PCNA 1:6,000 (sc-56) for nuclear extracts. At the end of the incubation, the primary antibody dilutions were removed, and the membranes were washed for 30 min with TBS-Tween 20, and the secondary antibody anti-murine-HRP (Thermo Scientific) was added for 1 h. At the end, membranes were washed in TBS and developed with Super Signal West Pico plus (Thermo Scientific) following the vendor’s instructions. Images were captured using the Fusion Fx Imaging System (Vilber Lourmant) and processed with the Evolution capt software.

### Tumorigenic assays

A total of 12 × 10^6^ cells of each of the established cell cultures were resuspended in 100 μl of RPMI-1640 and inoculated into the sub-scapular area of athymic Nu/Nu mice to evaluate for tumorigenesis. Tumor growth was evaluated at 6 weeks, after which the mice were euthanized and the tumor was resected for pathological analysis. These animals had free access to water and standard diet H2916 (Harlan, Indianapolis, IN).

### Housing and diet: Normocaloric vs. hypercaloric

The protocol for animal experiments was approved and registered at the research and ethics committee of the Instituto Nacional de Ciencias Médicas y Nutrición Salvador Zubiran (INCMNSZ) with registration number 1549 (377). Nu/Nu female mice were kept in the animal facility of the Instituto Nacional de Ciencias Médicas y Nutrición Salvador Zubirán under a protocol approved by the local animal ethics committee. After weaning, the animals were kept under an inverted schedule of 12 h light/12 h darkness in a room at 30°C with free access to water and to AIN-93 normo-caloric diet (20) (Bio-Serv, Flemington NJ) or to an inhouse-prepared high fat/high sugar diet (HFSD) (21). Every 100 g of pelleted diet contained 0.3 g L-cysteine, 0.25 g coline bitartrate, 1 g vitamins, 5 g cellulose, 3.5 g minerals, soy oil, 9 g starch, 11.4 g maltodextrin, 21.3 g sucrose, 24 g casein, 21.88 g lard, and 0.00133 tert-butylhydroquinone (TDHQ) (22). Animal weight was recorded every other day, and NMR was performed every month. Animals were used for the tumorigenesis assay once they reached a weigh of 20 g. Both groups received an implant of 20 × 10^6^ cells from the DSG-BC2 cell lines as described in tumorigenic assay. After 6 weeks, the animals were euthanized, and a necropsy was performed to recover tumors. C57BL/6 female mice were used for comparison of the effect of the hypercaloric diet (HFSD); these animals were kept under an inverted schedule of 12 h light/12 h darkness in a room at room temperature with free access to water and to AIN-93 normo-caloric diet (Bio-Serv, Flemington NJ) or to an inhouse prepared HFSD.

### Evaluation of body mass composition by NMR

Body mass composition was analyzed as previously described using an EchoMRI™ nuclear magnetic resonance system (21). Experimental animals were immobilized in restriction cylinders, and NMR signals were captured according to the vendor’s specifications. Animals were briefly exposed to a low-intensity electromagnetic field (0.05 T) for 2 min. Data processing provided lean and fat tissue values in grams; these data could be corrected with the total weight of each animal to express percent body fat or lean mass.

### Statistical analysis

The patient sample size was evaluated according to a Bernoulli type assay with the following formula for failure and success: *p ± z _α/2_ √(pq/n)*, where p was the probability of success. It was established to be 0.75, as 0.25 or 25% of the cell cultures would not develop, corresponding to q*. z _α/2_* was the probability that the confidence interval would contain μ, which was 95% or *z*=0.025. For n, or the number of explants needed, 32 patients were needed to establish a confidence interval of (0.60–0.90), such that 60%–90% of cell cultures would ultimately develop. All *in vitro* assays were conducted in triplicate and repeated at least in two or three independent experiments. Statistical significance in these data was established using Student’s t-tests and ANOVA, considering statistical significance with a p-value <0.05 (*) or a p-value <0.01 (**).

## Results

### Establishment of breast cancer cell lines based on BMI

This study was designed to establish breast cancer cell lines based on patients’ BMI to be able to determine if breast cancer that develops in a patient with a BMI of 18–25 kg/m^2^ has the same biological properties as the one that develops in a patient with obesity (>30 kg/m^2^). Tumor explants were obtained from patients with breast cancer and processed to generate cellular colonies attached to the bottom of tissue culture plates. Different methods were evaluated to determine the best way to generate primary cultures enriched in tumor cells (**Figure 1**). After surgical tumor removal, the pathologist removed small tumor samples (2–5 mm) and placed them in 50-ml sterile tubes with growth medium. Tumor samples were cut into small sections and attached to the surface of a dry Petri dish as described in *Materials and methods*. In the following 2 weeks, fibroblasts, recognized by their flat extended fusiform morphology, began to sprout out of the explants. Within the next 2 weeks, tumor cell colonies could be detected sprouting out of the explants alongside fibroblast proliferation. Tumor cells presented an ovoid or cuboid morphology with large nuclei, typically with two nucleoli and with filipodia and lamellipodia. We designed a cooperative sub-protocol between surgeons, pathologists, medical oncologists, researchers, and nurses that allowed efficient transfer from the operating room to the tissue culture facility reducing the time from the biopsy procurement to tumor clonal proliferation per explants from 2–3 months to 2–3 weeks.

Different methods were tested to obtain cell cultures enriched in tumor cells. The central problem was the elimination of fibroblasts from the primary cultures, which was solved using differential adhesion efficiencies. Cell populations that had emerged from tumor explants were detached using trypsin/EDTA when the Petri dish reached 80% confluence. After centrifugation, the supernatant was removed, and the pellet was resuspended in growth medium. Fibroblasts were allowed to adhere for 20 and 40 min, respectively, and the supernatant enriched in tumor cells was transferred to a new plate. In some instances, we used a cloning ring, specifically when tumor cell colonies grew in areas clearly separated from fibroblasts. To obtain the tumor cell colonies *via* cloning rings, the rings were sealed with silicone grease and placed around the colony. Tumor cells inside the ring were removed using trypsin/EDTA and placed into a separate Petri dish. An alternative approach implied digesting the tumor fragments with type II collagenase (300 U/ml) for 30 min at 37°C, incubating for 20 min in the incubator. The resulting digestion mixture was centrifuged and the pellet resuspended with growth medium and placed in two wells using differential centrifugation of the supernatant at 45, 100, and 200×*g*, respectively. This method was not efficient, as although tumoral fragments were disaggregated, the different cell populations including tumor cells, fibroblasts, epithelial cells, and adipocytes could not be separated. The faster proliferation rate of fibroblasts led to cultures dominated by fibroblasts within 7–10 days. The few tumor cells that were able to proliferate generated isolated colonies. The attempt to remove fibroblasts by scrapping proved to be inefficient. Hence, after adhering a tumor explant and collecting the cell populations that emerged within 2–3 weeks, the differential cell adhesion and ring cloning proved to be the most effective way to generate primary tumor cell cultures free from fibroblasts; *ergo*, they were both utilized in subsequent elaboration of primary tumor cell cultures (23–26).

We were able to establish two triple-negative breast cancer cell lines, one derived from a patient with normal weight (BMI < 25 kg/m^2^) and the second from a patient with obesity (BMI > 30 kg/m^2^), corresponding to explants 5 and 7. It is important to mention that the explant number 5, from which later the DSG-BC1 cell line was derived, came from a 59-year-old patient with ductal invasive breast cancer and a BMI of 21.9 kg/m^2^. Menses began at 14 years and menopause at 47. She had four pregnancies, all resulting in live births. She denied the use of contraception and had only a history of lipoma removal in 2000. The tumor immune phenotype was initially ER+ (92%), PR+ (95%), and HER2-negative tumor. During the late stages of establishing the primary culture, we observed a transformation into a triple-negative phenotype cell culture. With respect to explant number 7, from which later the DSG-BC2 cell line was derived, it came from a 52-year-old patient with invasive ductal carcinoma and a BMI of 31.5 kg/m^2^. Menses began at 11 years and menopause at 48. She had two pregnancies, both resulting in spontaneous abortions. She had no comorbidities. The tumor was a triple-negative immune phenotype, and the cell line preserved the same triple negative throughout the study. To be able to correlate findings related to differences in breast cancer in patients with obesity versus normal weight, it was essential to compare tumors with the same immune phenotype. We therefore present two triple-negative cell lines DSG-BC1 (BMI = 21.9) and DSG-BC-2 (BMI = 31.5).

### Immunophenotyping

**Figure 2:**
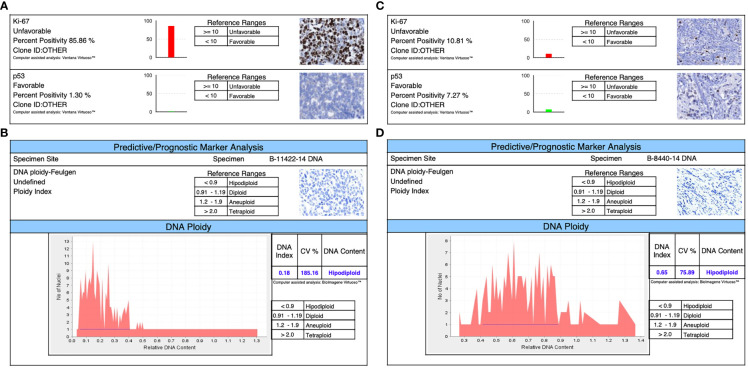
Immunophenotyping shows in both triple-negative lines: **(A)** for DSG -BC1, a Ki67 of 85.85% and a TP53 of 1.3%. **(B)** The ploidy of DSG-BC1 revealed a hypodiploid DNA content. **(C)** For DSG -BC2, a Ki67 of 10.81% and a TP53 of 7.27%. **(D)** The ploidy of DSG-BC2 revealed a hypodiploid DNA content.

Immunohistochemistry was performed using the Ventana BenchMark Ultra platform (Roche) using the following pre-diluted antibodies: anti-estrogen receptor (clone SP1, rabbit monoclonal, Roche), anti-progesterone receptor (clone 1E2, rabbit monoclonal, Roche), and anti-Her2 (clone 4B5, rabbit monoclonal, Roche). The stained slides were evaluated by a board-certified pathologist with extensive experience on breast pathology and immunohistochemistry using the current ER/PR and Her2 CAP/ASCO guidelines, in relation to the previously mentioned importance of the comparison of tumors with the same immune phenotype. Both cell lines were confirmed as triple negative as seen in **Figures 2A–D** (27, 28).

### Proliferation, migration, and invasion

**Figure 3:**
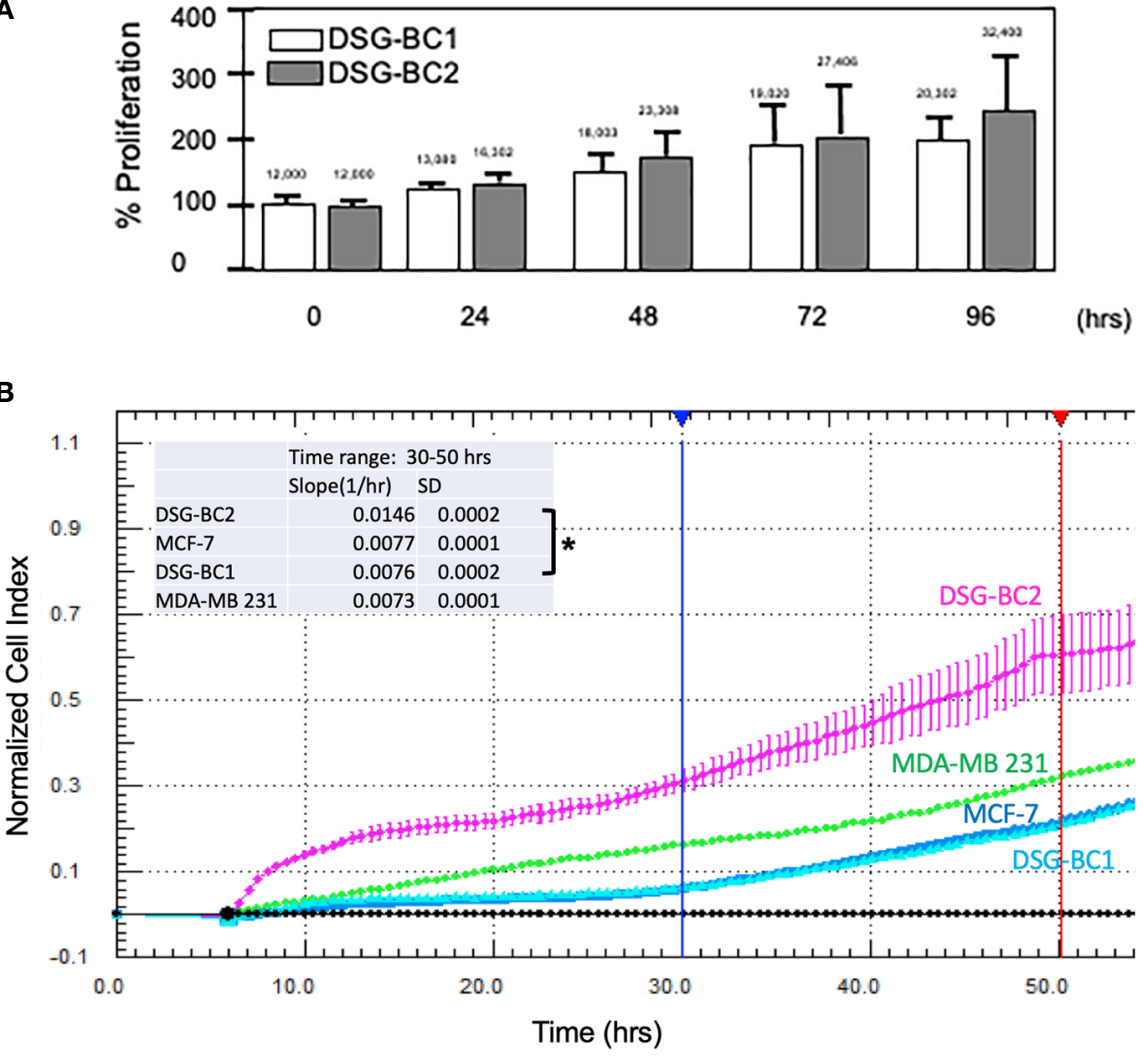
Breast cancer cells derived from a patient with obesity display a higher proliferation rate than the one derived from a patient with normal weight. **(A)** Direct cell counts every 24 h. Numbers above each bar represent the average cell number. **(B)** Normalized cell index measured every 30 min between 0 and 5 h. The inset represents the slope (change in normalized cell index/h) between 30 (blue vertical lines) and 50 h (red vertical line) of continuous growth. In panel **(B)**, MCF-7 and MDA-MB-231 cell lines were included as reference in cell growth. Experiment performed in triplicates; values represent average +/− standard deviation of the mean, n = 3. Vertical bracket indicates comparison between the two newly described cell lines, p value < 0.05 (*).

In an initial proliferation assay, the increase in cell number was analyzed by direct count with a hemocytometer (**Figure 3A**). Despite having plated the same initial number of cells, DSG-BC2 always related a higher cell count. By 72 h, it had 44% more cells than DSG-BC1. We also evaluated the proliferation rates in early time windows and compared them with the proliferation rates of the MDA-MD-231 and the MCF-7 cell lines. Cell proliferation during the first 50 h after plating was evaluated as an increase in impedance as described in *Materials and methods*. DSG-BC2 was able to grow 1.92 times faster than DSG-BC1 (**Figure 3B**). On the other hand, DSG-BC1, MCF-7, and MDA-231 presented very similar growth rates (0.077, 0.76, and 0.73 normalized cell index units/h). In both assays, we observed that the cell line DSG-BC2, derived from a patient with high BMI, presented a higher proliferation rate. The proliferation assays conducted lead to the understanding that DSG-BC2, the cell line of breast cancer and obesity, showed greater rates of proliferation in the three 2D systems assays in comparison to the cell line without obesity. Although this is seen *in vitro*, it may correlate to aggressive tumor growth *in vivo* in obese patients.

**Figure 4:**
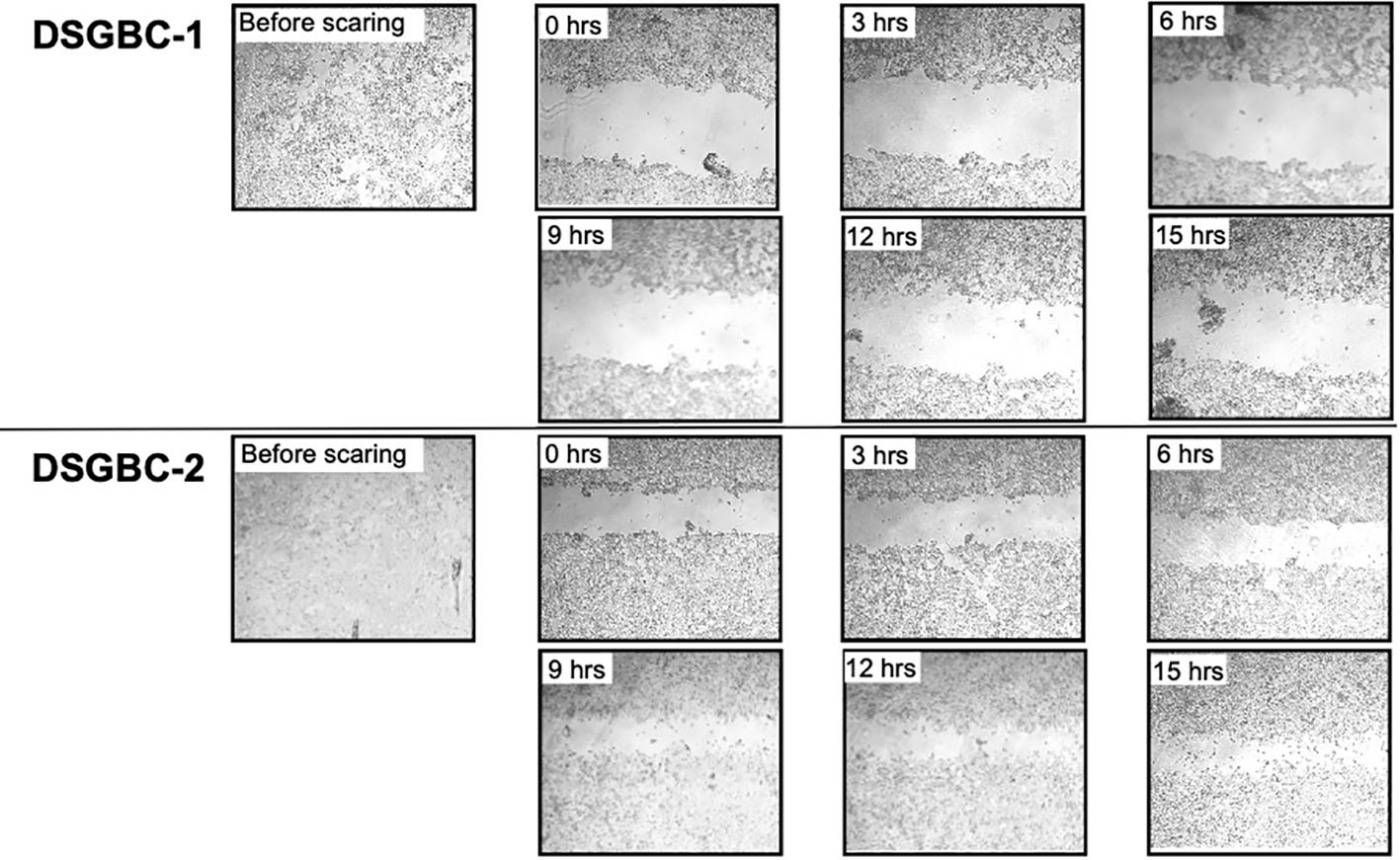
Breast cancer cells derived from a patient with obesity are faster in closing the space in a wound healing assay than the one derived from a patient with normal weight. Wound healing assay. Cells were plated to reach confluence within 24 h as described in *Materials and methods*. The invasion of the cell-free area was recorded every 3 h at the indicated time points. The cell-free areas were analyzed by Image J.

Petri dishes containing confluent cultures of DSG-BC1 and DSG-BC2 were scraped with a sterile pipet tip to create linear “wounds.” The separation of the remaining cell edges left a cell-free surface. After 24 h, filipodia and lamellipodia were observed to be emerging from the edges. The wound closing index was estimated with a total close at 72 h for DSG-BC1 and a total closing index at 96 h for DSG-BC2 (data not shown). Since within the timeframe of 72 h cell duplication might contribute to the reduction in free surface, we evaluated the reduction in free surface every 3 h from T0 to T15 hours after generating the linear wounds (**Figure 4**). The **Table 1** shows that the average reduction in free surface was 1.6 times faster in the DSG-BC2 cell line than in the DSG-BC1 cell line. The time frame for wound healing followed every 24 h is consistent with a faster proliferation of DSG-BC2 compared to DSG-BC1, correlating as well to the proliferation assays previously mentioned. The increase in cells in the open space generated when generating the wound follower every 3 h is less likely to be affected by cell division and can be considered to evaluate cellular movement. Hence, the DSG-BC2 breast cancer cell that arose in an obese environment can be seen to “move” faster than its counterpart.

**Table 1:** The table presents the average reduction in area (-D Area) every 3h, the bottom line presents the average of the 3 intervals.

		DSGBC1		DSGBC2	
		Speed -Δ Area/3hr		Speed -Δ Area/3hr	
	Interval	Avg	Std	Avg	Std
	3 to 6 h	10,977	12,877	29,056	17,460
	6 to 9 h	20,780	17,811	50,388	30,820
	9 to 12 h	25,334	18,659	13,427	12,286
					
Average interval					
-Δ Area/3h		19,030	3,122	30,957	9,564
	Fold	1.00		1.63	

Data represent average +/- standard deviation of the mean, n = 8.

**Figure 5:**
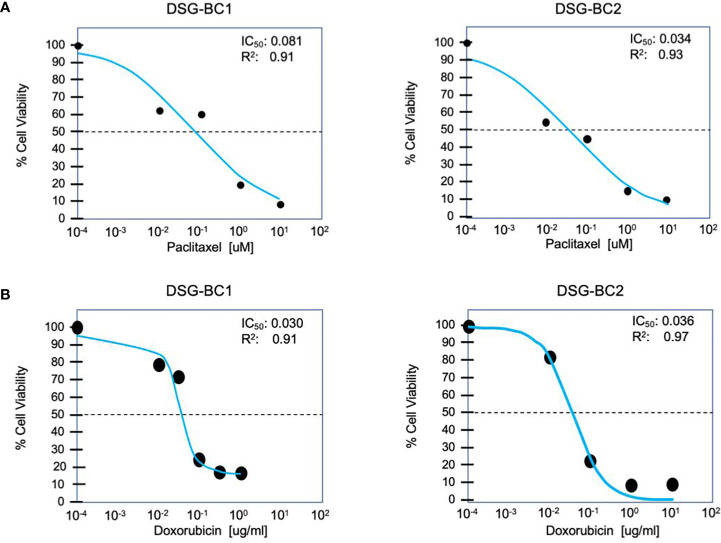
Breast cancer cells derived from a patient with obesity display lower chemosensitivity to paclitaxel compared to breast cancer cells derived from a patient with normal weight. Cytotoxicity assay for paclitaxel **(A)** and doxorubicin **(B)** after treatment for 48 h. n = 3.

Both DSG-BC1 and DSG-BC2 where exposed to increasing doses of paclitaxel to estimate IC_50_ after 48 h of treatment. The dose required for IC_50_ in both cell lines was within the same order of magnitude; nevertheless, the cell DSG-BC2 line, derived from the patient with high BMI, required 2.4 times more paclitaxel to reach IC_50_ (**Figure 5A**). The morphological changes during a time course of 96 h when treated with the IC50 of paclitaxel confirmed approximately 50% cell death after 48 h and 100% cell death after 96 h (**Figure 5B**). In the same way, both cell lines were exposed to increasing doses of doxorubicin to estimate IC_50_ after 48 h of treatment. In this case, both cell lines had the same IC_50_ of 0.3 μg/ml for doxorubicin (**Figure 5B**). The cytotoxicity assays for the cell lines show that DSG-BC2 needs higher doses of chemotherapy to be able to achieve similar results in terms of IC_50_ than DSG-BC1. In other words, we observed how breast cancer generated in obesity needed a greater dose of chemotherapy *in vitro* to achieve IC_50._

**Figure 6:**
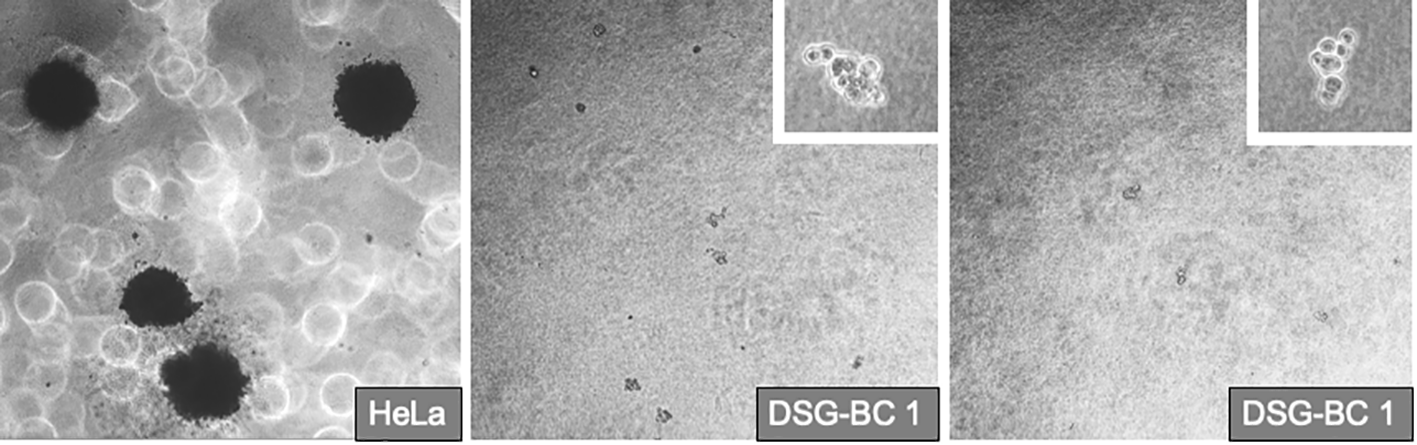
Breast cancer cells derived from patients with obesity or normal weight are not able to promote deformation of 3D colonies in soft agar. Even after 2 1/2 weeks after plating in the DSG-BC1 or DSG-BC2 in soft agar no 3D colonies could be detected only isolated cell aggregates could be seen (insets with 100× magnification). In comparison, HeLa cells generated multiple 3D colonies (left image with 40× magnification). n= 6.

Transformed phenotype and tumorigenic potential of tumor cells correlate with their ability to form 3D colonies when grown in soft agar and with their capacity to invade collagen matrixes. Their commitment in the epithelium–mesenchymal transition (EMT) is also indicative of their aggressiveness. We tested the ability of the DSG-BC1 and DSG-BC2 cell lines to grow by forming 3D colonies in the soft agar using HeLa cells as a positive control. The figure (**Figure 6**) shows that 2 weeks after initiating the cultures, HeLa cells were able to generate 3D colonies. In contrast, none of the two cell lines DSG-BC1or DSG-BC2 produced any 3D colonies, only isolated groups of a few cells with no indication of mitosis could be detected. The lack of 3D colonies in both cell lines suggests a low tumorigenic potential.

**Figure 7:**
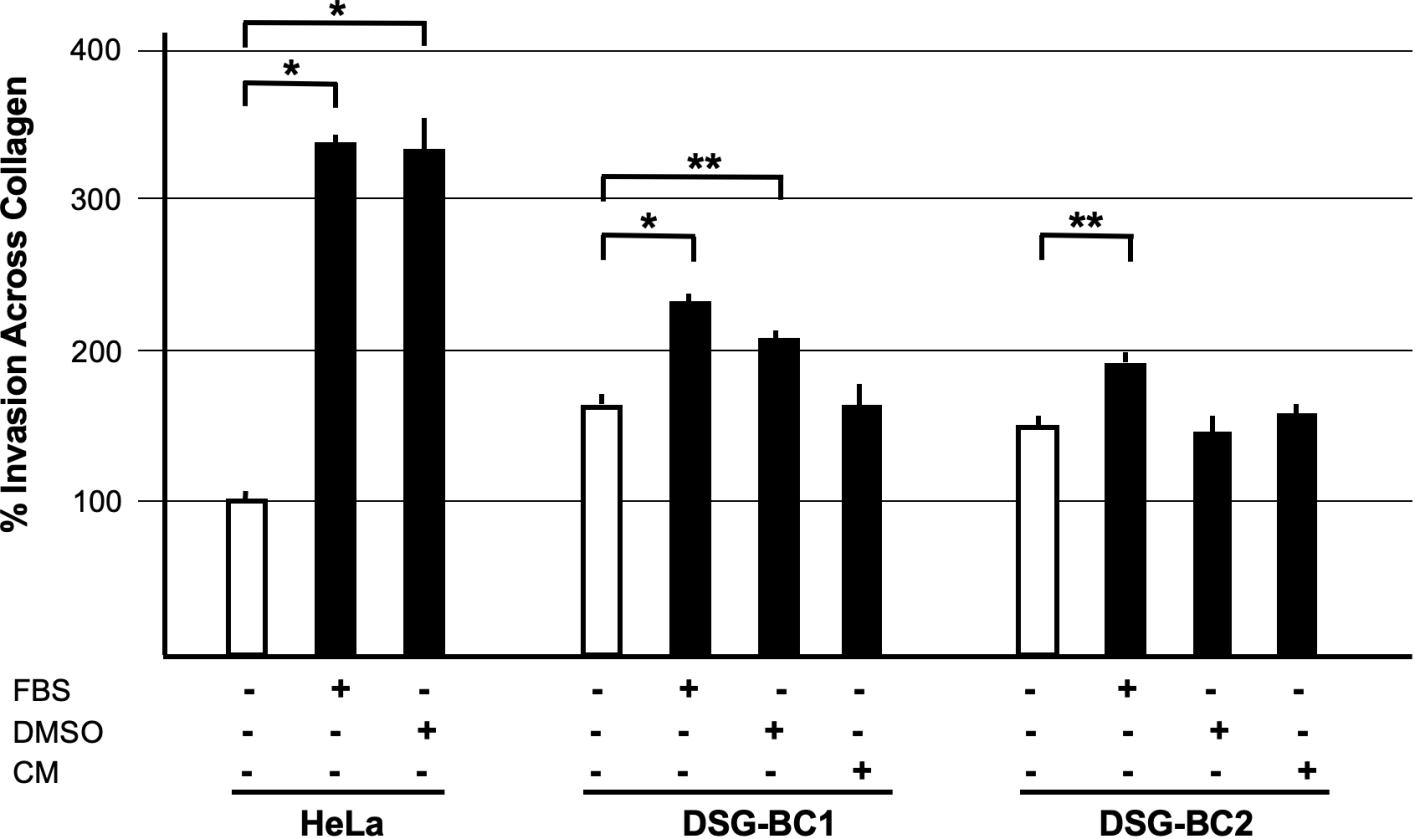
Breast cancer cells derived from a patient with obesity presented a higher invasion potential with respect to those derived from patient with normal weight. Invasion assay through collagen filled 8-μm pores. Cells were plated and, after adhesion, starved for 12 h before stimulation with the indicated attractants in the lower chamber as described in *Materials and methods*. HeLa cells were used as a positive control; 10% FBS supplemented growth medium was used as basal attractant in the lower chamber. Invasion of HeLa cells with this condition was considered 100% increase. Positive controls for invasion were 20% FBS (FBS), 80 nM PMA (PMA), or a 1:1 dilution of CM of each cell line. n=3. Brackets indicate comparisons with p value < 0.05 (*) or a p value < 0.01 (**).

The ability to migrate across the extracellular matrix is an essential hallmark for invasion and metastasis. We used Boyden chambers with 8-μm pores filled with collagen to test the invasive capacity of the two cell lines DSG-BC1and DSG-BC2 using HeLa cells for comparison. **Figure 7** shows that both DSG-BC1and DSG-BC2 were able to invade a collagen matrix with a 63% and 42% higher efficiency compared to HeLa cells. The use of 20% FBS in the lower chamber as attractant led to a further increase in invasion in both cell lines: 67% for DSG-BC1and 43% for DSG-BC2 compared to an increase in 238% in HeLa cells. The use of 80 nM PMA in the lower chamber as attractant increased invasion only in DSG-BC1 (47%), while in HeLa cells, PMA increased invasion (234%). In comparison, HeLa cells showed a stronger response to PMA with a 234% increase in invasion. Finally, we also tested a 1:1 dilution of the conditioned media of each cell line (CM) as attractant. CM had no effect on either DSG-BC1and DSG-BC2. These results suggest that both DSG-BC1and DSG-BC2 have the ability to invade collagen matrix, indicative of an aggressive phenotype.

**Figure 8:**
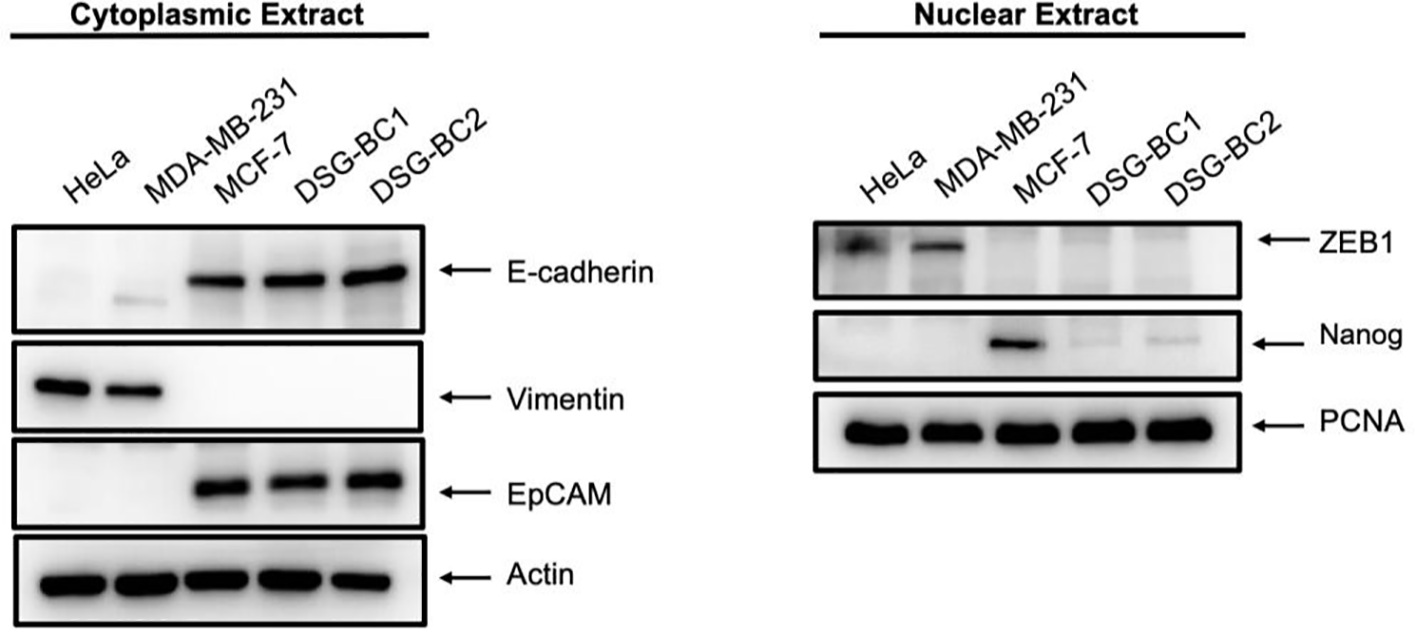
Breast cancer cells derived from patients with obesity and normal weight display a strong expression of epithelial markers and a weak and partial expression of mesenchymal markers. Western blot in cytoplasmic (left panel) or nuclear extracts (right panel) were performed as described in materials and methods. 40 ug of total protein were separated using SDS-PAGE and after electro-transfer membranes were probed with the indicated antibodies against EMT markers. Antibodies were diluted as described in material and methods. Actin was used as a loading control for cytoplasmic extract analysis, and PCNA served as loading control for nuclear extracts. Representative image of 3 independent experiments.

Transformation and tumorigenic potential require EMT; therefore, we analyzed the pression of EMT-molecular markers in both DSG-BC1 and DSG-BC2 and in HeLa, MDA-MB-231, and MCF-7 cells used for comparison. **Figure 8** shows the cytoplasmic expression of E-cadherin, EPCAM, and vimentin in cytoplasmic extracts and the expression of ZEB1 and Nanog in corresponding nuclear extracts. Both newly established cell lines DSG-BC1 and DSG-BC2 displayed a strong signal for epithelial markers, the cell adhesion molecules E-cadherin and EPCAM. In contrast, the mesenchymal-specific intermediate filament vimentin was absent in both cell lines. Mesenchymal master regulatory transcription factors ZEB1 and Nanog are weakly expressed in the nuclear extracts of both DSG-BC1 and DSG-BC2 cell lines. MCF-7 cells also expressed a strong signal for epithelial markers E-cadherin and EPCAM, but no vimentin. MCF-7 cells displayed a strongest signal for Nanog. In contrast, HeLa and MDA-MB-231 cells displayed a strong expression of vimentin and ZEB1 but had no Nanog expression. The strong expression of epithelial markers in both DSG-BC1 and DSG-BC2 suggests that the EMT is biased towards the epithelial phenotype. Nevertheless, the weak expression of ZEB1 and Nanog suggest that both cell lines have the potential to initiate the EMT.

### Endothelial cell activation

**Figure 9:**
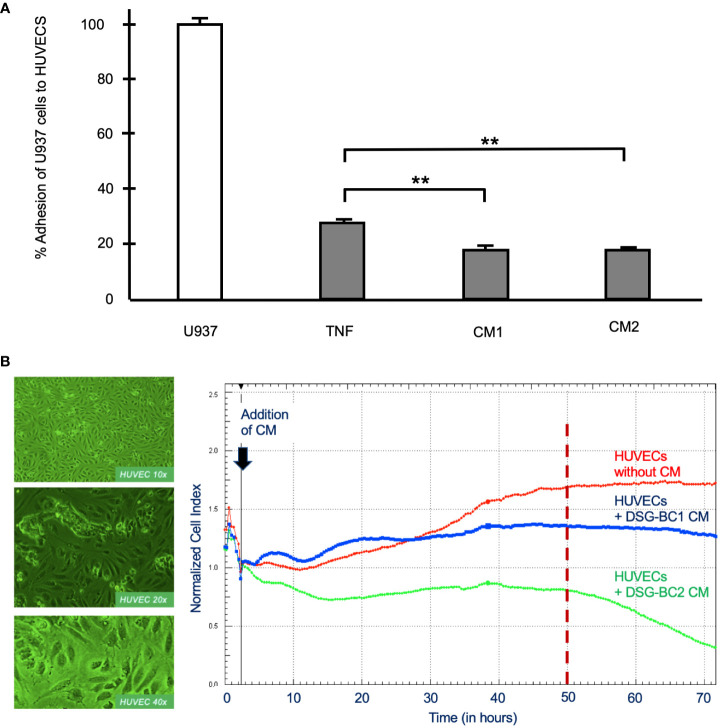
Breast cancer cells derived from patients with obesity or normal weight secreted similar amounts of soluble factor capable promoting endothelial cell activation. **(A)** % Adhesion of 3H-thymidine-labeled U937 cells to HUVECs treated for 3 hours with CM from DSG-BC1 or DSG-BC2 as described in materials and methods. 100% corresponds to the amount of radioactivity in 250,000 U937 cells (white bar). Normalized amount of radioactivity bound in HUVECS treated with TNF (10 ng/ml) or with 1:10 dilution of the CM from DSG-BC1 or DSG-BC2 (gray bars), n = 3. Brackets indicate comparisons with p value < 0.05 (*) or a p value < 0.01 (**). **(B)** Left panel: micrographs of endothelial cell monolayers at the indicated magnifications, taken before adding CM. Right panel: representative change in impedance in monolayers of HUVECs treated with a 1:10 dilution of CM of the indicated cell lines. CM were prepared as described in materials and methods and added 48 hours after plating (black arrow). Impedance was measured every 30 minutes as described in materials and methods. CM: conditioned media from the indicated cell type.

Malignancy is related to the secretion of a variety of soluble factors secreted by tumor cells to increase important elements in carcinogenesis, tumor proliferation and metastasis such as increasing vascular permeability and cellular adhesion, phenotypic changes seen in activated endothelial cells. We therefore collected conditioned medium (CM) from DSG-BC1 or DSG-BC2 and processed it as described in *Materials and methods* before testing their effect on the activation of primary human umbilical vein endothelial cells (HUVECs). The CM prepared from DSG-BC1 contained 39.0 μg protein/ml, compared to 19.0 μg protein/ml present in the CM from DSG-BC2. Endothelial cells treated with TNF (10 ng/ml) were able to bind 27 +/− 2% of the added U937 monocytes, while the treatment with CM from DSG-BC1 or DSG-BC2 promoted the adhesion of 18 +/− 3% and 16 +/− 2% of the added U937 cells, respectively (**Figure 9A**). The binding of the U937 monocytes to the HUVEC endothelial cells aimed to show how the soluble factors released by tumor cells into the CM can lead to an activated endothelial phenotype that could contribute to a metastatic phenotype.

In addition, we also tested the effect of the CM from both cell lines on the permeability of monolayers of HUVECs measuring impedance and so epithelial permeability (**Figure 9B**). Impedance increased with time in untreated control HUVECs and stabilized 50 h after addition of control medium (red trace in **Figure 9B**). In the presence of the CM from DSG-BC1, impedance increased in two waves and was markedly reduced 20 h after its addition (blue trace in **Figure 9B**). In the presence of the CM from DSG-BC2, impedance fell immediately after its addition (green trace in **Figure 9B**). After 50 h of treatment, the CM from DSG-BC1 had reduced impedance by 20%, while the CM from DSG-BC2 reduced impedance by 52%. Impedance of endothelial cell monolayers is an indirect measure of epithelial permeability known to be affected during metastasis. The reduction in impedance induced by the CM of both cell lines indicates the presence of bioactive secreted products able to reduce endothelial permeability. The fact that the cell line derived from a patient with higher BMI is suggestive of a more aggressive phenotype. With this, an inference on its metastatic potential may be postulated.

### Sensitization of breast cancer cell growth to estradiol (E2) by chronic exposure to leptin

**Figure 10:**
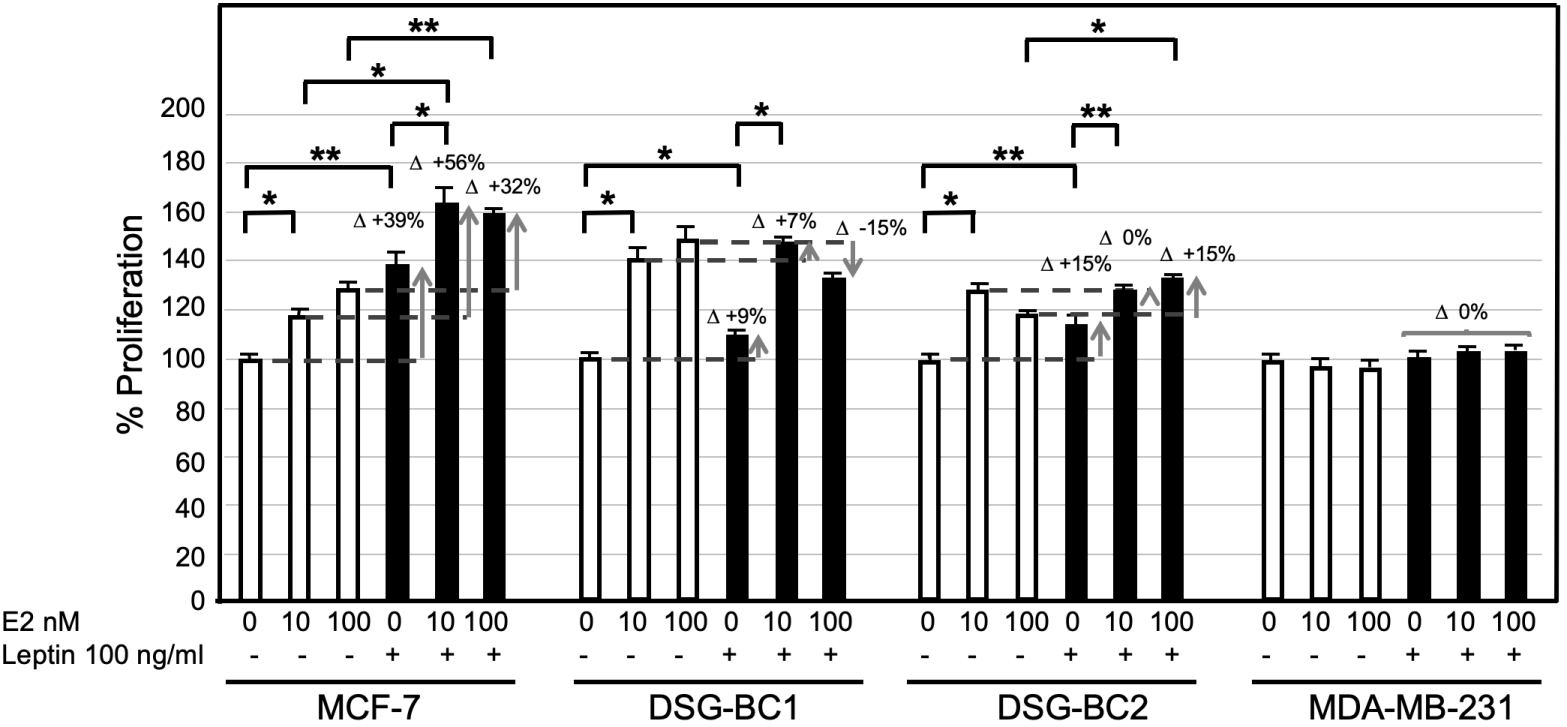
Breast cancer cells derived from an obese patient are sensitized to estrogen-proliferation by chronic exposure to leptin. The indicated 4 breast cancer cell lines were cultured as described in materials and methods: treated for 4 days with leptin (100 ng/ml) changed to medium without phenol red and supplemented with lipid free serum for 24 hours before the final stimulation with estradiol (E2) at the indicated concentrations for 48 hrs. Control cultures received no leptin stimulation (empty bars). Sensitization is depicted at the increase in proliferative response (D+). Values are the average of triplicates +/- standard deviation; n = 3. Brackets indicate comparisons with p value < 0.05 (*) or a p value < 0.01 (**).

Among the different adipokines liberated by adipose tissue in obesity, leptin has been postulated to contribute to breast cancer tumorigenesis and progression. Treating the newly established cell lines DSG-BC-1 and DSG-BC-2 with leptin (100 ng/ml) for 48 h did not affect cell proliferation (data not shown). Leptin is known to have a wide spectrum of biological effects including the sensitization of breast cancer to estradiol-dependent proliferation (14). MCF-7, DSG-BC1, DSG-BC2, and MDA-MB-231 where pretreated with leptin for 5 days and then challenged with 0, 10, and 100 nM E2 for further 48 h. The histogram in **Figure 10** shows that 5-day continuous exposure to leptin sensitized MCF-7 to E2, leading to a 56% increase when treated with 10 nM E2 and a 32% when treated with 100 nM E2. These results are in agreement with previous reports (14). Interestingly, the cell line DSG-BC-2 derived from a patient with obesity presented a 15% increase in proliferation when treated with 100 nM E2, while in DSG-BC-1, breast cancer cells isolated from a patient with normal BMI, pretreated with leptin, led to a 15% decrease in proliferation when stimulated with 100 nM E2. MDA-MB-231 cells displayed no response to E2 or to leptin sensitization. This result suggests that despite the fact that both DSG-BC1 and DSG-BC-2 have a triple-negative phenotype, they express low levels of ER. Our results also indicate that the ER signaling pathway in breast cancer cells derived from an obese patient can be sensitized by leptin, an adipokine normally present in patients with increased dysfunctional adipose tissue.

### Leptin activates the JAK2/STAT3/AKT signaling pathway in DSG-BC-1 and DSG-BC-2 breast cancer cells

**Figure 11:**
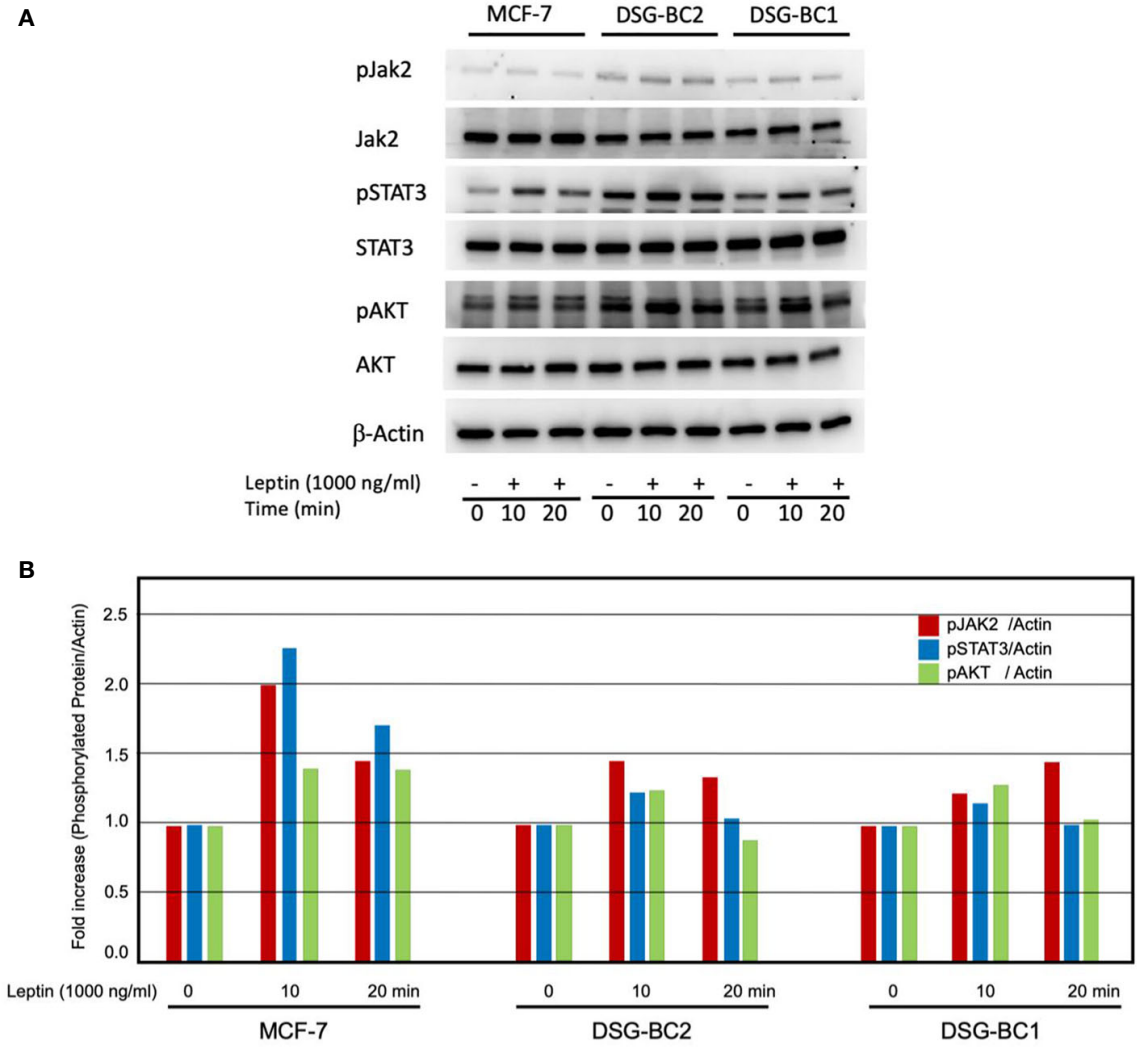
Western blot analysis revealed rapid activation of the JAK/STAT3/AKT signaling pathway in response to leptin both in DSG-BC-1 and DSG-BC-2 breast cancer cell lines. Cells (4 × 10^5^) were plated in six-well plates. After 24 h, cell extracts were prepared at the indicated time points (0, 10, and 20 min) of leptin stimulation (1,000 ng/ml), as described in *Materials and methods*. **(A)** Total cell extract (30 μg) was separated in 7.5% SDS-PAGE. Western blot analysis for total JAK2 and phosphorylated JAK2 (pJAK2), total STAT3 and phosphorylated STAT3 (pSTAT3), and total AKT and phosphorylated AKT (pAKT). **(B)** Histogram of the normalized signals of the indicated phosphoproteins of the panel **(A)** Phosphoprotein signals were corrected with the signal of beta actin (β-actin); images were digitalized and analyzed using the public ImageJ software.

The sensitization effects suggest that both DSG-BC-1 and DSG-BC-2 cells can respond to leptin. We tested if exposure to a leptin could activate phosphorylation of signaling molecules linked to leptin receptor activation. We treated both DSG-BC-1 and DSG-BC-2 cells and MCF-7 cells with 1,000 ng/ml leptin for 10 and 20 min and evaluated the change in the phosphorylation state of JAK2, STAT3, and AKT through Western blot analysis. **Figure 11A** shows a representative result of the Western blot analysis, and the histogram in **Figure 11B** presents the normalized intensity of the phosphorylated signaling proteins. As previously reported, in control MCF-7 cells, leptin induced a transient increase in phosphorylation both of JAK2 and STAT3 in the first 20 min of stimulation, while AKT phosphorylation presented a steady 35% increase. Interestingly the DSG-BC-2 cell line derived from an obese patient displayed a stronger response compared to the DSG-BC-1 cell line derived from a patient with normal BMI. These results strongly suggest that both cell lines present functional leptin receptors and that the JAK2/STAT3/AKT signaling pathway in the cell derived from obese patients presents a stronger response to this adipokine.

### *In vivo* experiments

**Figure 12:**
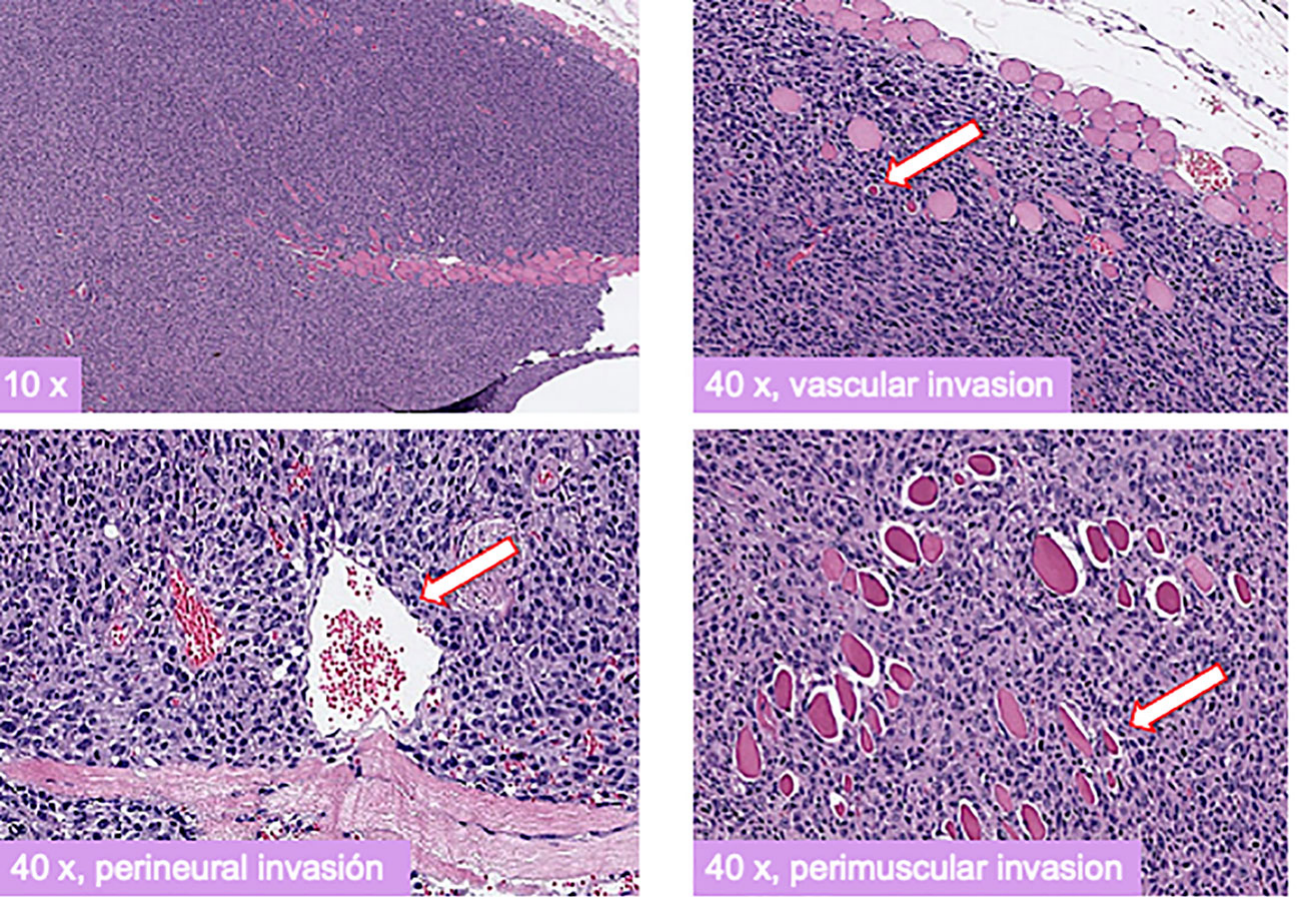
Xenotransplants of the breast cancer cells derived from a patient with obesity displayed a more aggressive tumorigenic and invasive activity compared to those derived from a patient with normal weight. Histopathology of H&E-stained slides from tumors generated 3 weeks after implantation of cell from the DSG-BC2 cell line. Images were taken at the indicated magnifications. White arrows indicate transversal section of a vascular structure (upper right panel), transversal section of a nerve (lower left panel), and transversal section of a muscle fiber (lower right panel). Samples were processed as described in *Materials and methods*.

Seven athymic Nu/Nu female mice, fed with a standard diet, were inoculated with 10 × 10^6^ cells of each cell line, DSG-BC1 and DSG-BC2, totaling 14 mice. Seven days after the inoculation, tumor growth was seen in every mouse. The largest tumor was observed in mouse 1 at 14 days with a 10 × 2 mm tumor on the right subscapular area and mouse 2 at 10 days with a 4 × 5 mm tumor on the right subscapular area, both originating in mice inoculated with the DSG-BC2 cell line (29–31). Immunohistochemical analysis for ER, PR, and HER2 confirmed a triple-negative immune phenotype for both DSG-BC1 and DSG-BC2. The histopathological analysis of the tumors in hematoxylin and eosin-stained slides revealed perivascular, neural, and muscular invasion (**Figure 12**), which was not observed in comparison to tumors generated by DSG-BC1. Although it was corroborated that both tumors had the same triple-negative immune phenotype, DSG-BC2 showed a greater infiltrative nature with the vascular, neural, and muscular invasion, most likely due to the aggressiveness that the metabolic environment in which it was created confers.

**Figure 13:**
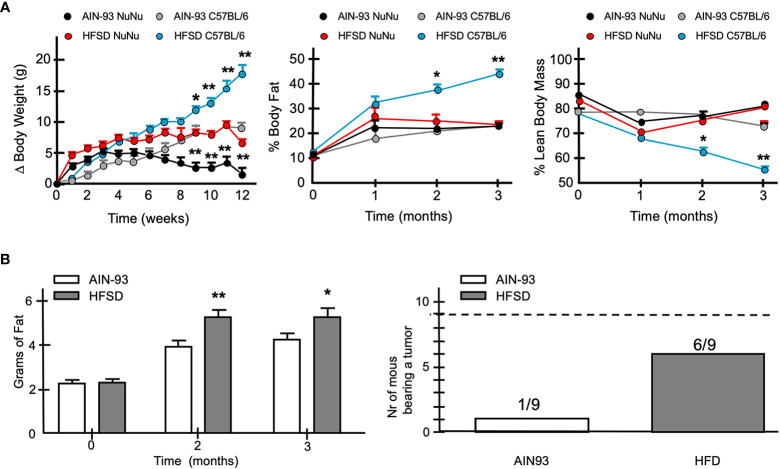
When fed a hypercaloric diet Nu/Nu female mice mimic a mild overweight condition and favor more frequent tumor development of DSG-BC-2 xenotransplants. Tumorigenesis in female Nu/Nu mice fed with normocaloric (AIN93) o hypercaloric (HFSD) diet from weaning to the end of the tumorigenic assay. Animals were kept as described in materials and methods until they reached a weight of 20 g. **(A)** Increase in weight once they reached 20 g in Nu/Nu or C57B/6 female mice receiving AIN93 or HFSD (left panel), % of body fat (central panel) and % lean body mass (right panel). **(B)** Total weight estimated by NMR of Nu/Nu mice fed with the AIN93 or HFSD (left panel). Number of the mice bearing tumors from a total of 9 mice that received 20 x 106 cell of the DSG-BC2 that were fed with either AIN93 or HFSD (right panel); n = 9, p value < 0.05 (*) or a p value < 0.01 (**).

To evaluate the influence of an obesogenic *in vivo* environment, we fed Nu/Nu female mice with a defined normo-caloric rodent diet (AIN93) or a high fat – high sucrose diet (HFGD) as previously described. While HFGD promoted a significant increase in weight in normal C57BL/6 mice, it had a more modest effect in Nu/Nu mice (**Figure 13A**). Nu/Nu mice fed with the normo-caloric diet AIN93, had on average 5 g lower weight than those fed with HFGD. Despite this difference, no significant change in body mass composition was observed after 3 months (**Figure 13A**). However, when fed with HFGD, the fat tissue estimated by NMR was higher compared to those fed with AIN93 (**Figure 13B**). This difference was identified as an increase in subcutaneous fat accumulation. A group of nine Nu/Nu female mice fed with either diet was inoculated with 20 × 10^6^ cells from the DSG-BC2 cell line, and tumor growth was monitored as described in *Materials and methods*. While in the group of mice fed with AIN93, only one mouse developed a tumor mass. In the group fed with HFSD, six animals developed tumor growth (**Figure 13B**). The unexpected observation that Nu/Nu mice appear to have a higher basal energy expenditure compared to C57BL/6 mice could explain the marginal effect of HFSD in developing overweight. In addition, metabolic analysis of the sera from both groups of Nu/Nu revealed that with AIN93, glucose metabolism was the main source of aerobic metabolism, while ketone body metabolism was the primary source of energy with HFSD (data not shown). It is possible that these differences are due, at least in part, to the lack of coat in animals of the Nu/Nu strain. This would explain the large difference in the increase in weight when both strains where fed with the hypercaloric diet HFSD (**Figure 13A**). The greater tumorigenic ability of DSG-BC2 in mice fed with HFSD could pertain to possible growth requirements from the original tumor, which was generated in a metabolically obese environment.

## Discussion

Obesity has become an openly recognized risk factor not only for the development of breast cancer but also its aggressiveness and recurrence (15, 32, 33). Women with breast cancer and obesity have a worse disease-free and overall survival despite appropriate local and systemic therapies (34). The importance of this epidemiological comorbidity in Mexico is relevant worldwide (35).

In addition, systemic chemotherapy is less effective, even when dosed appropriately on the basis of actual weight. A central mechanism by which obesity stimulates cancer progression is through chronic, low-grade inflammation in adipose tissue. Counter measures such as exercise in the AIM trial (36) seek to alleviate and reduce systemic inflammation, metabolic diseases, possibly leading to interrelated biomarkers involved in the associations between obesity, exercise, and breast cancer prognosis. Notwithstanding, we currently are in a discovery phase where factors within obesity, adipocytes, and inflammation factors change longstanding paradigms related to cancer treatment. Mechanisms underlying the obesity–cancer relationship are poorly understood (37, 38). Patients who are obese require special treatment considerations for adequate management and optimal therapeutic efficacy (39).

An important element in the connection with breast cancer and the obese setting is the adipose tissue microenvironment with its complex association to inflammatory factors that promote tumor growth, invasion, and metastasis (40). One of the most influential factors is the adipokine leptin, which is an important molecular mediator of the obesity–breast cancer axis. Increase adiposity, and thus increased leptin secretion, promotes tumor cell proliferation as an independent factor for neoplastic aggressiveness, with functions strengthened through interactions with multiple oncogenes, growth factors, and cytokines (41, 42). It has even been proposed as a novel target in therapeutic strategies for breast cancer given the rise in immune therapy and its proinflammatory mediation (43, 44). The evaluation of leptin levels among the two cell lines is an important step needed in their further characterization.

The link between obesity and breast cancer is clearly a multifactorial and dynamic process that includes pre- versus post-menopausal condition, relative abundance of visceral versus subdermic adipose tissue, and ethnicity (45–47). In post-menopausal women, adipokines also contribute to the risk of breast cancer. While circulating levels of leptin have a strong direct correlation to the incidence of breast cancer, high levels of adiponectin have a protective effect (48, 49). This altered adipokine balance has been postulated to drive the increased expression of aromatase in obese adipose stromal cells leading to an increase in biotransformation of androgenic substrates into estrogens (49, 50), and coincides with higher incidence of ER+ breast cancer in obese patients (46, 48). Other hallmarks of malignancy such as motility, invasion, and anti-tumor immunity are promoted by cancer-associated adipocytes (51, 52). In breast cancer linked to obesity, the non-genomic estrogen receptor crosstalk with the PI3K/Akt and MAPK pathways is increased (53), and the genomic methylation state is altered and related to survival expectancy (54). The response to leptin presented in **Figures 6**, **7** indicates that both cell lines have leptin receptors, although specific proof is still necessary by Western analysis and RT-PCR. In particular, activation of the JAK2/STAT3/AKT signaling pathway further indicates the functionality of these receptors. The fact that a more robust phosphorylation could be observed in DSG-BC-2 derived from an obese patient is in agreement with the postulated role of leptin in the promotion of breast cancer cells in obese patients. The sensitization to E2 is particularly interesting, since it could reveal a crosstalk between ER expression, ER signaling and leptin signaling. Induction of functional ERs has been reported in human breast cancer cells, promoting conversion of ER− to ER+ cells amenable to antiestrogen therapy (55, 56). Nevertheless, a more careful analysis of the signaling events and changes in gene expression must be performed to confirm this view.

Human cell lines have been derived from specific subtypes of breast cancer and have served to define the cell physiology of the corresponding breast cancer subtypes (57). The luminal-A MCF-7 cell line, established in 1973 at the Michigan Cancer Foundation, was derived from a pleural effusion from a 69-year-old white patient with recurrent disease (26, 58). Through functional assays model, cell lines such as MCF-7 cells have led to the identification of a complex epigenetic mechanism where a PAD2-dependent histone H3R26 citrullination facilitates ER transcription activation (59). This cell line has also served to identify the participation of miR23a in the induction of EMT and metastasis in luminal A breast cancer (60). The availability of breast cancer cell lines derived from patients with defined BMI and metabolic alterations related to overweight and obesity will allow to describe and unravel functional alterations linked to these comorbidities (61–63). The triple-negative MDA-MB-231 cell line was derived from a pleural effusion from a 51-year-old Caucasian woman with a metastatic mammary adenocarcinoma with a marked increase in chromosome number, between 65 and 69 (64); nevertheless, no information is available.

Starting with solid tumor biopsies and using standard explant cell culture techniques combined with differential cell adhesion and ring cloning, we established two cell lines DSG-BC1 and DSG-BC2 that display a triple-negative molecular phenotype. DSG-BC1 was derived from a 59-year-old patient with ductal invasive breast cancer and a BMI of 21.9 kg/m^2^. DSG-BC2 was derived from a 52-year-old patient with invasive ductal carcinoma and a BMI of 31.5 kg/m^2^.

A comparison of basic biological characteristics revealed differences in their proliferating potential and in their ability to move into a cell-free space in a wound healing assay. They presented minor differences in their sensitivity to paclitaxel but were equally sensitive to doxorubicin. Their secretoma was able to activate endothelial cells inducing similar pro-adhesive phenotypes but displayed a different ability to increase endothelial permeability. The behavior of all these *in vitro* assays of the DSG-BC2 cell line suggests a more aggressive phenotype with a faster growth rate and motility, lower sensitivity to paclitaxel, and higher ability to induce endothelial permeability. Even more, in the tumorigenic assay in Nu/Nu mice fed with a normo-caloric diet, the DSG-BC2 cell line presented vascular, neural, and muscular invasion compared with the DSG-BC1 cell line. Nevertheless, the most significant difference was their tumorigenic ability when implanted in Nu/Nu mice fed with diets with different caloric content, trying to mimic eutrophic versus adipogenic *in vivo* metabolic conditions. The DSG-BC2 cell line generated tumors with higher frequency (6/9) when implanted in mice fed with a hypercaloric diet (HFSD) compared to a lower frequency (1/9) in mice fed with the normocaloric diet (AIN93). This result suggests that the obese condition of the patient where this tumor developed left growth requirements imprinted that favored tumor growth in the obesogenic environment created by the hypercaloric diet.

Taken together, the proliferation potential, motility, *in vitro* invasive capacity, the expression of mesenchymal markers, and *in vivo* tumorigenicity and invasive capacity suggest that tumor cells generated in an obese individual are more aggressive than their counterparts that arise in patients with normal weight.

## Conclusion

We present a novel approach to study the comorbidity of obesity and breast cancer by establishing cell lines derived from patients with breast cancer and with different BMIs. We also report that in addition to differences in the biological characteristic analyzed *in vitro*, these cells present differences in their tumorigenic capacity when implanted in mice fed with normo- versus hyper-caloric diets. This new model will allow functional studies and the analysis of altered molecular mechanisms under the comorbidity of obesity and breast cancer.

## Data availability statement

The raw data supporting the conclusions of this article will be made available by the authors, without undue reservation.

## Ethics statement

The animal study was reviewed and approved by research and ethics committee, Instituto Nacional de Ciencias Médicas y Nutrición Salvador Zubirán.

## Author contributions

DSG, RG-C, AC, and AZ-D contributed to conception and design of the study. DSG and AZ-D wrote sections of the manuscript. CL performed the pathological analysis on the original tumor explants and the mice specimens and wrote sections of the manuscript. JB, AM-A, ES, GC, and CR are the oncologic surgeons involved in patient selection and history, as well as obtaining tumor explants. JV contributed to the establishment of the cell lines. AV, NT, AT, LBG, MC, and JG contributed with the evaluation of obesity in the cell lines, contributed with the evaluation of the cell lines in obese mouse models, designed the high fat and sugar diets, wrote sections of the manuscript. All authors contributed to manuscript revision, read, and approved the submitted version.
